# Redefining the tumor microenvironment with emerging therapeutic strategies

**DOI:** 10.32604/or.2024.055161

**Published:** 2024-10-16

**Authors:** SULING XU, XIAO LI, WENXUE MA

**Affiliations:** 1 Department of Dermatology, The First Affiliated Hospital of Ningbo University, Ningbo, 315010, China; 2 Department of Interventional Therapy, National Cancer Center/National Clinical Research Center for Cancer/Cancer Hospital, Chinese Academy of Medical Sciences and Peking Union Medical College, Beijing, 100021, China; 3 Department of Medicine, Sanford Stem Cell Institute and Moores Cancer Center, University of California San Diego, La Jolla, CA 92093, USA

**Keywords:** Tumor microenvironment (TME), Cancer immunotherapy, Cytokine targeting, Extracellular vesicles (EVs), Adoptive cell transfer (ACT)

## Abstract

The environment surrounding a tumor, known as the tumor microenvironment (TME), plays a role in how cancer progresses and responds to treatment. It poses both challenges and opportunities for improving cancer therapy. Recent progress in understanding the TME complexity and diversity has led to approaches for treating cancer. This perspective discusses the strategies for targeting the TME, such as adjusting networks using extracellular vesicles to deliver drugs and enhancing immune checkpoint inhibitors (ICIS) through combined treatments. Furthermore, it highlights adoptive cell transfer (ACT) therapies as an option for tumors. By studying how components of the TME interact and utilizing technologies like single-cell RNA sequencing and spatial transcriptomics, we can develop more precise and efficient treatments for cancer. This article emphasizes the need to reshape the TME to boost antitumor immunity and overcome resistance to therapy, providing guidance for research and clinical practices in precision oncology.

## Introduction

Exploring the terrain of the tumor microenvironment (TME) reveals a battleground for cancer treatment, where cytokines and other substances play a double role in the battle against cancer. Recent studies have shed light on the diverse nature of the TME, presenting both challenges and opportunities for creating cancer therapies [1–3]. Xu et al. [4] provide an overview of our knowledge and treatment approaches to the TME. This article expands on their findings, delving into the potential impacts of progress and future paths for TME-focused treatments.

## Heterogeneity of the TME in Different Solid Tumors

Xu et al. stress the diversity of the TME and how it influences treatment outcomes [4]. Recent studies using single-cell RNA sequencing have uncovered a range of cell types in the TME, highlighting the importance of treatment strategies [5–7]. For instance, single-cell RNA sequencing has uncovered subgroups of cells with specific functional statuses. This includes T cells that show levels of immune checkpoint proteins and macrophages that support tumor growth while exhibiting immunosuppressive characteristics [8]. Additionally, these studies have identified rare cell populations, such as cancer stem cells (CSC) and immune-suppressive fibroblasts, which play critical roles in tumor progression and resistance to therapy [9,10]. Using transcriptomics, scientists have mapped out how different cell types are organized within the TME, offering insights into how cellular interactions impact cancer progression and response to treatment [11–13].

The composition and components of the TME can vary significantly across different types of solid tumors, influencing carcinogenesis and therapeutic responses in diverse ways. For instance, the TME in breast cancer often contains high levels of cancer-associated fibroblasts (CAFs) and immune suppressive cells, such as regulatory T cells (Tregs) and myeloid-derived suppressor cells (MDSCs), which contribute to tumor progression and metastasis [14,15]. In contrast, the TME in pancreatic cancer is typically characterized by a dense desmoplastic stroma, rich in fibroblasts and extracellular matrix (ECM) components, which creates a physical barrier to immune cell infiltration and drug delivery [16,17].

Glioblastomas exhibit a TME with significant infiltration of microglia and macrophages, which can support tumor growth and invasion through the secretion of immunosuppressive cytokines like transforming growth factor beta (TGF-β) and IL-10 [18]. Meanwhile, the TME in colorectal cancer often shows a prominent inflammatory component with pro-inflammatory cytokines such as IL-6 and transforming growth factor alpha (TNF-α), which can promote tumor cell proliferation and survival through the activation of the STAT3 and NF-κB pathways [19,20].

The TME in melanoma is notable for its high degree of immune cell infiltration, including T cells and dendritic cells (DCs), which can be targeted by immune checkpoint inhibitors. However, the presence of immunosuppressive factors and cells, such as PD-L1 expressing tumor cells and Tregs, can still limit the efficacy of these therapies [21,22].

Additionally, the TME in prostate cancer plays a critical role in its development, progress, and metastasis of prostate cancer [23]. It often has a unique metabolic environment, with altered lipid metabolism and the presence of adipocytes contributing to tumor growth and resistance to therapy [24]. The diversity in the cellular composition, cytokine profiles, and metabolic characteristics of TMEs across different solid tumors underscores the need for tailored therapeutic approaches that consider the specific TME context of each tumor type [25].

Recognizing this diversity is vital for developing targeted treatment. The intricate network formed by cells, stromal cells, and extracellular matrix components plays a crucial role in supporting tumor growth and evading immune surveillance. For instance, tumor-associated macrophages (TAMs) can have pro-tumorigenic or anti-tumorigenic functions based on their polarization status. Understanding the roles and interactions of these cells in the TME can guide the development of therapies that alter the TME to promote antitumor responses [1].

Furthermore, several unique physiological aspects of the TME present lucrative targets for TME-directed therapy. The reverse Warburg effect, wherein CAFs undergo metabolic reprogramming to support tumor cell metabolism, highlights the intricate metabolic interactions within the TME [26]. Hypoxia-induced reprogramming within the TME stabilizes hypoxia-inducible factors (HIFs), which promote angiogenesis, metabolic adaptation, and immune evasion [27]. Additionally, the role of prostaglandins and inflammatory cascades in the TME supports chronic inflammation and tumor progression, making them attractive targets for therapeutic intervention [28].

Advanced techniques like single-cell RNA sequencing and spatial transcriptomics are essential for mapping these variations and identifying specific cellular interactions and pathways that can be targeted to improve treatment outcomes [12,29].

**Figure 1:**
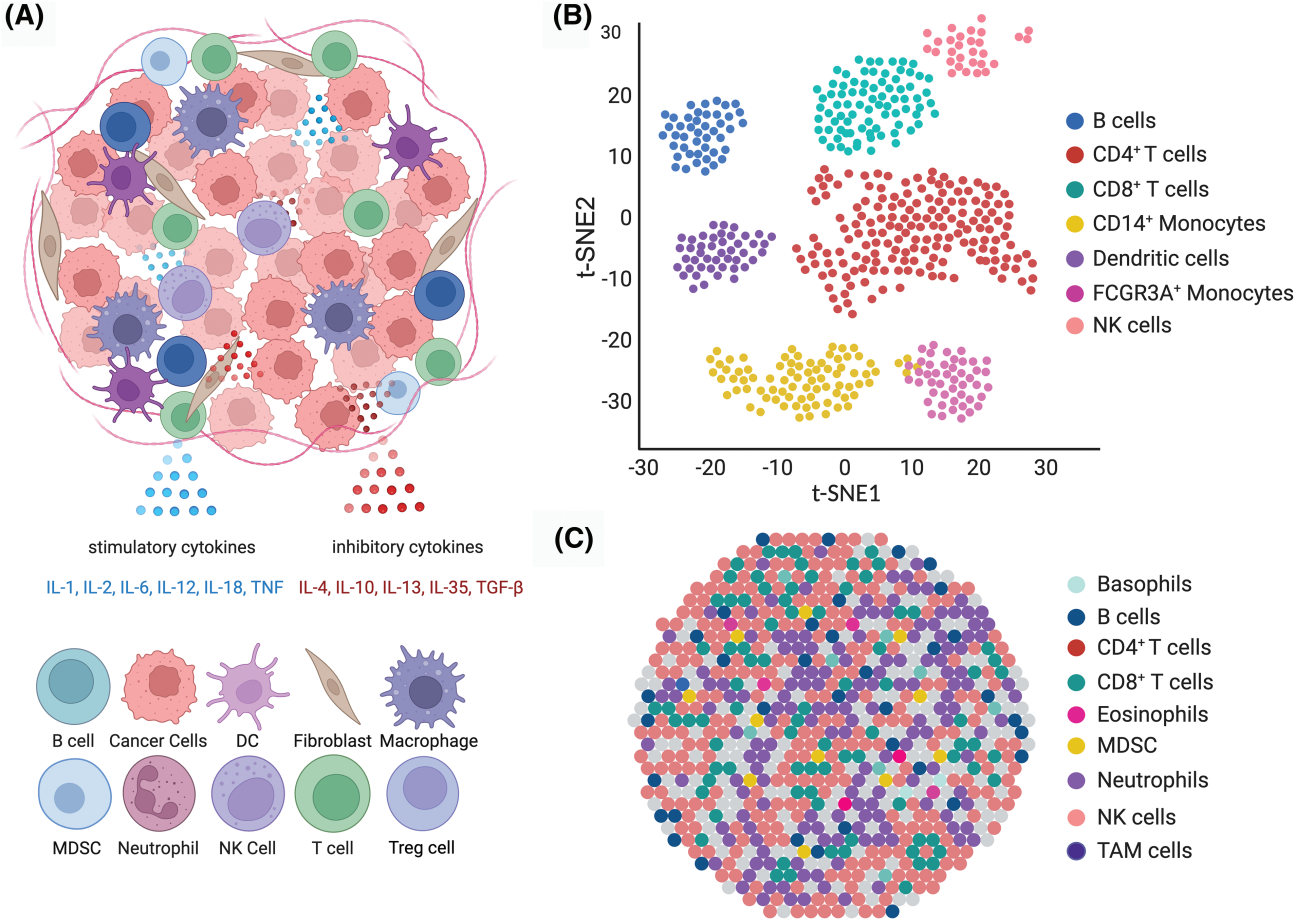
Diverse cell types and interactions within the TME.

Fig. 1 highlights the diverse cell types in the TME, their interactions, and the insights obtained from advanced sequencing technologies to illustrate these complexities and interactions. This illustration showcases the diverse nature of the TME, revealing a variety of compositions and interactions through single-cell RNA sequencing and spatial transcriptomics. The communication between these cells is facilitated by cytokines like IL-1, IL-2, IL-6, IL-12, and IL-18 (stimulatory), and IL-4, IL10, IL-13, IL-35, and TGF-β (inhibitory). Understanding these interactions is vital for developing targeted treatments that manipulate the TME to support antitumor responses. (A) Schematic representation of the cell types in the TME, including tumor cells, various immune cells (e.g., T cells, NK cells, B cells, DCs, monocytes), stromal cells (e.g., fibroblasts), and macrophages. Key cytokines such as TNF, IL-1, IL-6, IL-12, IL-18 (stimulatory), and TGF-β, IL-4, and IL-10 (inhibitory) mediate interactions between these cells, creating a network that facilitates tumor growth and immune evasion. (B) t-SNE plot illustrating the clustering of cell populations within the TME based on the data from single-cell RNA sequencing. Each point on the chart represents a cell, with color indicating the cell type, such as NK cells, CD8^+^ T cells, CD14^+^ monocytes, B cells, DCs, CD4^+^ T cells, and FCGR3A+ monocytes. (C) The spatial arrangement of cells in the TME is visualized through transcriptomics. The dot shows the spatial layout and potential interactions between various cell types within the tumor tissue.

## Targeting the Cytokine Network

Xu et al. delve into the functions of cytokines in the TME and their potential as targets for treatment [4]. Recent studies have uncovered that cytokines like interleukin (IL)-27 can either promote or hinder tumor growth depending on their situation [30,31]. Research suggests that IL-27 can stimulate responses against tumors by encouraging the development of CD8^+^ T cells, indicating its promise as a target [32–34].

Apart from IL-27, other cytokines such as IL-6, IL-10, and TGF-β also shape the TME [35,36]. For example, IL-6 fuels inflammation and aids tumor progression by activating signaling pathways like Janus kinase/signal transducers and activators of transcription 3 (JAK/STAT3) [37,38]. Efforts are underway to target these cytokines or their receptors to disrupt the tumor signals within the TME. Monoclonal antibodies directed at IL-6, or its receptor (IL-6R) have displayed results in animal models and are currently undergoing evaluation in clinical trials [36,39].

## Extracellular Vesicles in Cancer Therapy

Extracellular vesicles (EVs), including exosomes, are essential for communication between the cells in the TME. They help to transfer molecules and regulate immune responses [40,41]. Scientists discuss how to utilize EVs for therapy, which shows promise [4,42]. Advances in nanotechnology have led to drug delivery systems using exosomes, which can target tumor cells effectively while reducing systemic toxicity [4]. Recent research has shown that engineered exosomes can deliver small interfering RNA (siRNA) to target oncogenes, resulting in significant tumor regression in mouse models [43,44].

Researchers are also investigating using EVs as biomarkers for cancer diagnosis and prognosis [45–47]. The molecular content of EVs reflects the condition of the cells they derived from, making them valuable for non-invasive liquid biopsies [40]. For example, specific microRNAs or proteins found in circulating exosomes can offer insights into tumor development and response to treatment. Developing methods to isolate and analyze EVs from patient samples is essential for translating these discoveries into clinical applications [48].

## Immune Checkpoint Inhibitors and Combination Therapies

The advancements made by immune checkpoint inhibitors (ICIs), like pembrolizumab and nivolumab, have significantly changed cancer treatment [49,50]. However, Xu et al. point out that the effectiveness of these inhibitors may be hindered by the immune-suppressing characteristics of the TME [4]. Combined with agents such as TGF-β inhibitors that alter the TME, ICIs have shown synergistic results in animal studies, boosting cancer immune responses and enhancing treatment outcomes [51]. A couple of research studies have indicated that pairing pembrolizumab with a TGF-β inhibitor decreased tumor growth and increased survival rate in a mouse melanoma model [52,53].

Apart from TGF-β inhibitors, researchers are exploring combined approaches, like pairing ICIs with radiation therapy, targeted therapies, or oncolytic viruses, to improve the effectiveness of ICIs [54,55]. These combination strategies are designed to modify the TME by increasing the presentation of tumor antigens, decreasing cell populations, and bolstering the infiltration and function of effector T cells [56].

## Adoptive Cell Transfer Therapies

ACT therapies, like chimeric antigen receptor (CAR) T-cell therapy and tumor-infiltrating lymphocyte (TIL) therapy, have achieved impressive success in leukemia and are now being investigated for solid tumors [57]. Xu et al. [4] delve into the potential of boosting ACT effectiveness by adjusting the TME to support the longevity and function of transferred cells. Pairing CAR T-cell therapy with cytokine modulation to improve T-cell survival and proliferation within the TME has shown promise in animal studies [58].

A hurdle faced with ACT in solid tumors is the suppressive nature of the TME, which can hamper the infiltration and function of transferred cells [49,59]. Strategies to conquer these challenges include priming the TME with chemotherapy or radiation, genetically engineering T cells to express receptors that aid in homing to the tumor site and combining ACT with checkpoint inhibitors. These approaches aim to create a better microenvironment for the antitumor function of transferred cells [60].

## Challenges and Future Directions

While these new developments show promise, a couple of obstacles remain. Xu et al. [4] have pointed out the varied nature of the TME in different cancer types, emphasizing the need for a deeper understanding of the mechanisms that drive TME reprogramming. It is essential to develop biomarkers for profiling the TME to customize treatment for individual patients [61–63]. Future research efforts should concentrate on integrating omics approaches and advanced computational models to forecast responses and enhance treatment strategies [64]. Combining single-cell transcriptomics with spatial profiling of the TME can create a comprehensive map, identifying cellular interactions and signaling pathways for targeted intervention [12,65].

Given the changing nature of the TME, it is essential to monitor and adapt therapeutic approaches. Real-time imaging methods and non-invasive biomarkers must be developed to evaluate treatment responses accurately and adjust therapies promptly as needed [66]. For instance, liquid biopsies offer real-time insights into cellular changes within the TME, facilitating precise and adaptive therapeutic interventions [46].

Another emerging field of interest is understanding how the microbiome influences the TME. Research has shown that the gut microbiome can impact the effectiveness of cancer therapies, including immunotherapies [67,68].

The relationship between the microbiome and the TME has the potential to introduce approaches that influence the system’s response and improve the efficacy of current therapies. Additionally, there is a growing focus on understanding how metabolic changes in the TME play a role [69,70]. By targeting metabolic pathways like glycolysis and oxidative phosphorylation with the TME, we may disrupt the supportive environment of cancer cells and enhance antitumor immune responses [71].

**Figure 2:**
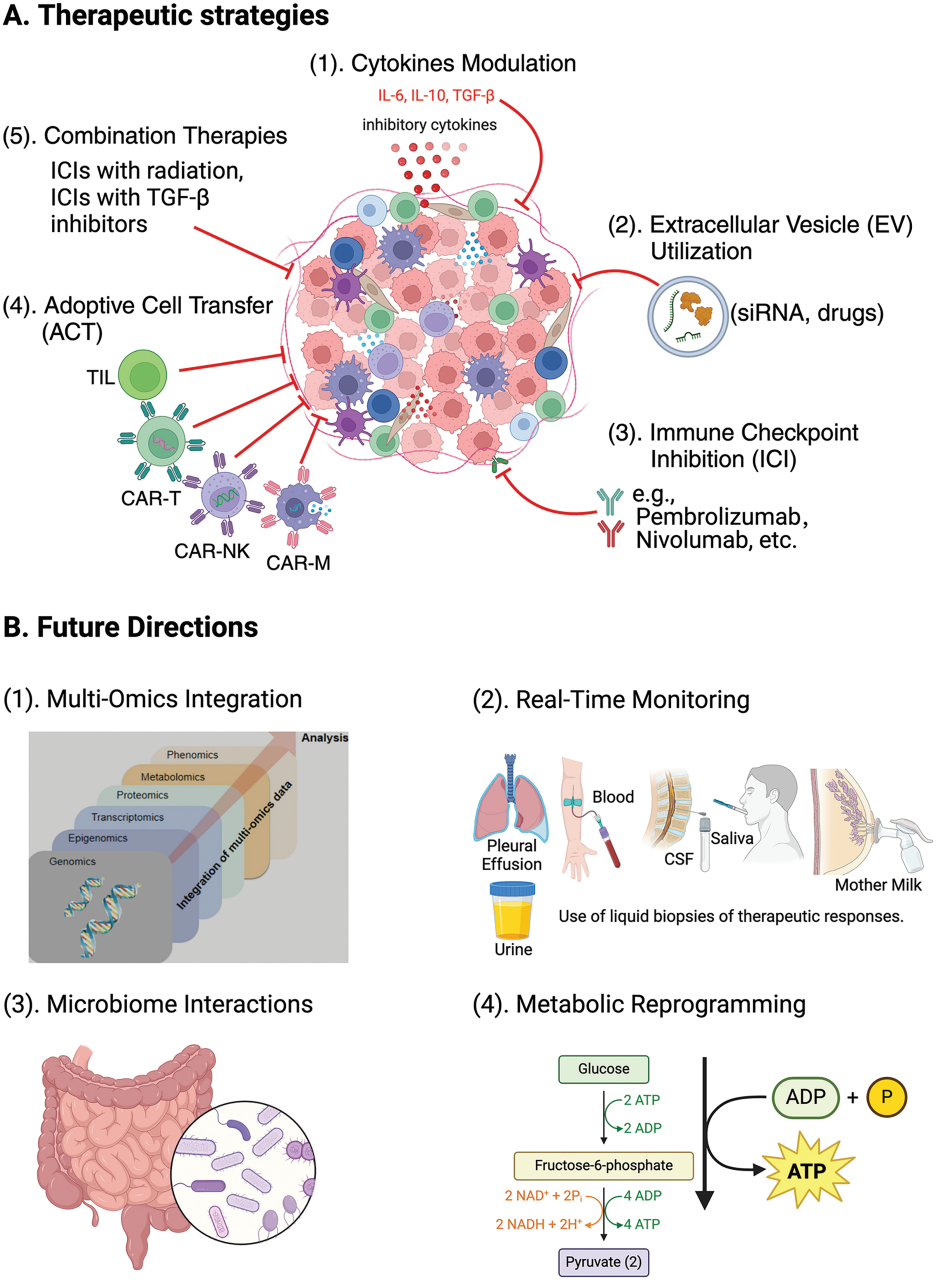
New therapeutic strategies targeting the TME.

To summarize the treatment approaches and their potential effects, we showcase Fig. 2 below.

Fig. 2A offers an overview of creative methods being developed to target the TME, highlighting treatment strategies like (1) adjusting cytokine levels (modifying cytokines such as IL-6, IL-10, and TGF-β to reprogram the TME and enhancing antitumor immune responses against tumor); (2) using EV for delivering of therapeutic agents like siRNA and medications directly to tumor cells; (3) inhibiting immune checkpoint molecules with inhibitors like pembrolizumab and nivolumab to increase T-cell activity against tumor cells; (4) employing CAR T-cell therapy; CAR-NK cell therapy; CAR-Macrophage cell therapy, and (5) TIL therapy to strengthen antitumor immunity through adoptive cell transfer methods, and integrating immunotherapy with other treatments. Fig. 2B illustrates the potential directions for future research in integrating immunotherapy with other treatment modalities (1) merging omics data (including genomics, transcriptomics, proteomics, epigenomics, metabolomics, and phenomics) to obtain a thorough insight into the TME and improve treatment strategies, (2) real-time tracking (using liquid biopsies including blood, urine, saliva, cerebrospinal fluid (CSF), pleural effusion, and breast milk to monitor treatment responses promptly and adjust therapies as needed, (3) examining microbiome interactions to understand how the gut microbiome impacts the immune response and the effectiveness of cancer treatment, and (4) targeting metabolic reprogramming pathways such as glycolysis and oxidative phosphorylation to disrupt the tumor-supportive environment and boost antitumor immune reactions. Understanding these methods and future directions is essential for advancing cancer treatments.

## Conclusion

The complex interaction of cytokines and other substances in the TME poses both challenges and opportunities for cancer treatment. Xu et al. provide an overview of the status of the TME and potential approaches to address it. Recent progress in understanding the nature of TME and exploring treatment strategies show promise in improving cancer therapy outcomes. Targeting TME to boost tumor immune response and tackle treatment resistance effectively is critical. Ongoing research efforts and innovative developments in this area are vital for maximizing the benefits of reshaping the TME and paving the way for a new era in precision oncology.

## Data Availability

Data sharing is not applicable to this article as no datasets were generated or analyzed during the current study.

## Abbreviations

ACT: Adoptive cellular therapy
CAF: Cancer-associated fibroblast
CAR: Chimeric antigen receptor
CSC: Cell stem cell
DC: Dendritic cell
ECM: Extracellular matrix
EV: Extracellular vesicle
ICI: Immune checkpoint inhibitor
IL-6: Interleukin 6
HIF: Hypoxia-inducible factor
IL-6R: Interleukin 6 Receptor
JAK/STAT: Janus kinase/signal transducers and activators of transcription
MDSC: Myeloid-derived suppressor cell
NK: Natural Killer
TAM: Tumor-associated macrophage
siRNA: Small interfering RNA
TIL: Tumor-infiltrating lymphocyte
TGF-β: Transforming growth factor beta
TME: Tumor microenvironment
Treg: Regulatory T cell
